# Assessing the impact of lentiviral vector integration on splicing of cellular genes at the genome-wide level

**DOI:** 10.1186/1742-4690-8-S2-O15

**Published:** 2011-10-03

**Authors:** Eugenio Montini, Jacopo Sgualdino, Daniela Cesana, Fabrizio Benedicenti, Stefania Merella, Simone Leo, Gianluigi Zanetti, Luigi Naldini

**Affiliations:** 1 San Raffaele Telethon Institute for Gene Therapy, Milan, Italy; 2 Center for Advanced Studies, Research, and Development in Sardinia, Pula, Italy

## 

Oncogenesis induced by insertional mutagenesis with gene therapy vectors occurs mainly by activation of proto-oncogenes found at or nearby the insertion site. This activation often occurs by an enhancer-mediated mechanism or by a process of splicing capture which generates chimeric transcripts comprising portions of vector and cellular mRNAs. Although the activation of oncogenes may be reduced by the use of self-inactivating design and moderate cellular promoters, how to reduce genotoxic splicing capture events and aberrant transcript formation triggered by vector integration is still unclear.

We developed a modified Linear Amplification-Mediated (LAM) PCR technique, named cDNA LAM PCR (cLAM-PCR), aimed at retrieving, from the whole transcriptome of LV-transduced cells aberrantly spliced mRNAs that contain lentiviral vector (LV) sequences fused with cellular transcripts in a high-throughput fashion. The sequences of cLAM-PCR products were obtained by 454 pyrosequencing and analyzed by dedicated high-throughput computational pipeline running in a computer cluster that use a dynamic analysis process composed by different steps based on a map-reduce parallelization model. Thus, chimeric LV-genome sequences are recognized, the nucleotide position of the fused sequence is identified (the splice site), and the remaining portion mapped on the appropriate genome assembly by BLAST.

We identified several established and previously unknown splice sites within the LV backbone that participate in the aberrant splicing process with variable efficiency. Results obtained with different LV designs show that integrated LVs can perturb the processing of cellular transcripts by interacting with the cellular splicing machinery and fusing with its own splice sites to cellular splice sites both upstream and downstream the integration site. So far, 70 different fusion transcripts could be identified in total, 84% of which were fused to known splice sites of gene exons, 6% were fused to uncharacterized cryptic splice sites located in introns and the remaining 10% were fused to genomic sequences not corresponding to any annotated gene. This analysis allows identifying also several different slice sites within the LV backbone that participated to the aberrabt splicing process. Quantitative PCR on different LV portions within the LV backbone allow measuring the relative contribution to the aberrant splicing process of each splice site identified. Interestingly, the amount of transcription occurring in regions outside the expression cassette reaches the 3% of the entire transgene expression.

The cLAM-PCR technique, coupled to high-throughput sequencing and the computational power of our specialized data analysis pipeline allows gaining insights into the biology of vector-mediated splicing alteration. Since this process could induce neoplastic transformation by the generation of aberrant oncogenic protein, its in-depth characterization is instrumental in the development of next-generation LV with a higher safety profile.

